# Asymptomatic pneumoperitoneum in pneumatosis coli: A misleading operative indication

**DOI:** 10.1016/j.ijscr.2020.03.042

**Published:** 2020-04-01

**Authors:** Marta Ribolla, Luigi Conti, Edoardo Baldini, Gerardo Palmieri, Carmine Grassi, Filippo Banchini, Maria Diletta Dacco’, Patrizio Capelli

**Affiliations:** a Department of Medicine and Surgery, AOU Parma, Via Gramsci 14, Parma, Italy; b Department of Surgery, AUSL Piacenza, Via Taverna 49, 29121 Piacenza, Italy

**Keywords:** Pneumatosis cystoides intestinalis, Pneumoperitoneum

## Abstract

**Introduction:**

Pneumatosis cystoides intestinalis (PCI) is a rare clinical pathology characterized by sub-mucosal and/or sub-serous cysts of free gas, forming cystic lesions usually ranging from 0.5 to 2.0 cm in size within the gastrointestinal tract. About 3% of patients with PCI develop complications such as pneumoperitoneum, intestinal volvulus, obstruction, or hemorrhage, these cases need immediate surgical intervention. Cyst rupture can produce peritoneal irritation and pneumoperitoneum.

**Presentation of case:**

A 65-years-old woman was admitted to the Emergency Department for epileptiform convulsions. Her medical hystory included epilepsy, diabetes, lichenoid dermatitis, hypothyroidism, severe cognitive impairment. Abdominal CT scan revealed a dilated large intestine with parietal pneumatosis from the appendix to the transverse colon associated to extensive pneumoperitoneum. The patient underwent emergency laparotomy which revealed the presence of gas within the wall of right and transverse colon and distension of great omentum. No resection was needed as normal blood supply to the bowel present.

**Discussion:**

Pneumatosis coli can be both asymptomatic or life-threatening condition associated to bowel infarction; this situation can mimic a bowel perforation causing pneumoperitoneum - that sometimes is a non-surgical pneumoperitoneum - and it could be a misleading indication to surgical exploration especially in the case of uncertain origin of a septic shock.

**Conclusions:**

We report a case of pneuomoperitoneum due to PCI. Surgical intervention was required for patient’s conditions and unclear origin of the sepsis.

## Highlights

• This case report highlights the importance of careful consideration of clinical and radiographic findings in the diagnostic and therapeutic approach to pneumoperitoneum especially when asymptomatic.
• The surgeon has to consider all the potential causes of non-surgical pneumoperitoneum.
• Decision-making process for surgical versus non-surgical management of pneumoperitoneum is crucial to avoid unnecessary surgery for the patient.

## 1 Introduction

This work has been reported in line with the SCARE criteria [1].

Pneumtosis cystoides intestinalis (PCI) is a rare clinical pathology characterized by sub-mucosal and/or sub-serous cysts of free gas, forming cystic lesions usually ranging from 0.5 to 2.0 cm in size within the gastrointestinal tract. PCI can be detected in all parts of the gastrointestinal tract and was first documented by Du Vernoy in 1783 [2], defined as primary PCI.

The term secondary PCI was termed by Koss in 1952 [3], who analyzed 213 pathological specimens and attributed 85% of the cases to a secondary disease [4,10,11]. It has a similar CT appearance of a perforated viscus [6]. The incidence of PCI was reported to be 0.03% in the general population [3], the mean age was 60.4 +/− 18.9 years without genders difference. Although most PCI are asymptomatic at diagnosis and incidentally detected on CT or during surgery, symptoms may include abdominal pain (79%) followed by nausea/vomiting (27%) and abdominal distension (19%) [5]. Most cases of pneumatosis cystoides are harmless, but due to difficulties in distinguishing it from perforated viscus and necrotizing enterocolitis, a large percentage of patients unnecessarily receive operative intervention. PCI can be a rare complication of systemic sclerosis characterized by accumulation of gas within walled-cysts. It is postulated to result either from excess hydrogen gas produced by intraluminal bacterial pressure of nitrogen within the intestinal wall (the bacterial theory) [7], or from the translocation of gas cysts through the layers of bowel wall as a result of high luminal pressure, intestinal obstruction, inflammatory bowel disease [14], ischemic bowel disease, gastroenteric tumor, anorectal surgery, bowel preparation or colonscopy (the mechanical theory) [7,12,13], or from pulmonary diseases that may result in pulmonary alveolar rupture and then produce a pneumomediastinum that dissects along the aorta and then along the mesenteric vessels to the bowel wall (the pulmonary theory) [15], or from malnutrition that can prevent the digestion of carbohydrates and increased bacterial fermentation in the intestine, producing large volumes of gas leading to distention and ischemia and subsequently the submucosal dissection of gas (the chemical theory or the nutritional deficiency theory) [16,17]. There have been also some reports on PCI associated with chemotherapy, hormonal therapy and connective tissue disease [[18], [19], [20]]. The more widespread use of diagnostic CT in recent years has led to increased recognition of this condition, a finding that also often raises concern for intestinal necrosis or perforation [8]. Pneumatosis cystoides intestinalis in patients with systemic sclerosis is a benign condition that generally resolves with bowel rest, antibiotics and supportive care [9]. About 3% of patients with PCI develop complications such as pneumoperitoneum, intestinal volvulus, obstruction, or hemorrhage, these cases need immediate surgical intervention. Cyst rupture can produce pneumoperitoneum and peritoneal irritation.

## 2 Case report

**Fig. 1:**
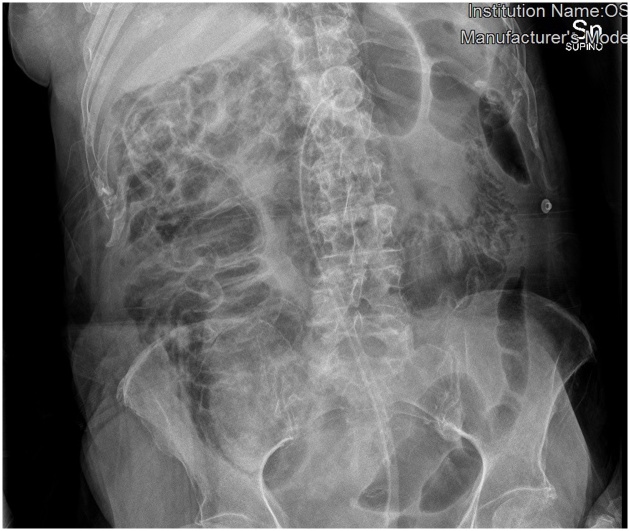
X-ray of abdomen shows important bowel distension.

**Fig. 2:**
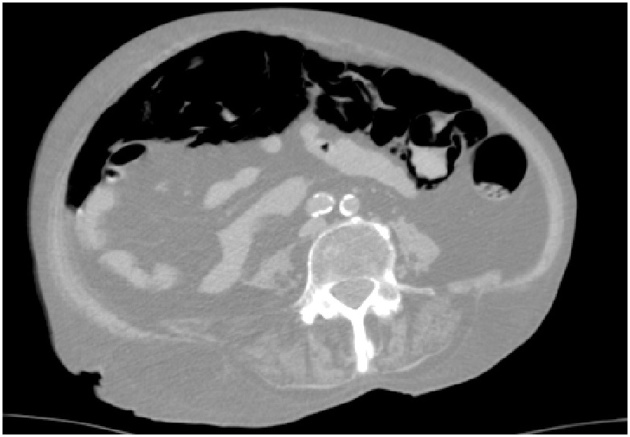
CT scan shows an extensive pneumoperitoneum.

**Fig. 3:**
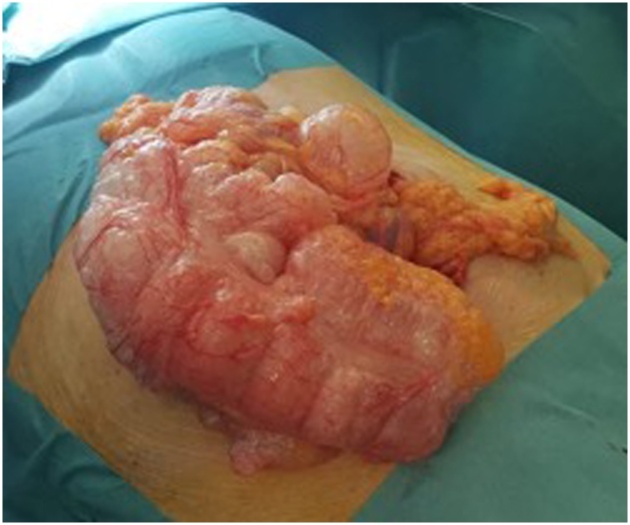
Intra-operatory finding of pneumatosis of the right colon.

A 65-years-old woman affected by diabetes, lichenoid dermatitis, hypothyroidism, severe cognitive impairment, epilepsy and PEG-bearer was admitted to the Emergency Department for incoming epileptic seizures. She had been previously hospitalized for respiratory failure caused by an ab ingestis episode (MRSA pneumonia), that needed a temporary tracheostomy. The patient was unresponsive, GCS 8. Laboratory tests revealed a severe leukocytosis (WBC counts 33.95 × 10^3^), blood acidosis (Ph 7.37, lactate 98 mg/dL, Base excess – 6.7 mmol/L), both signs of a sever septic shock and initial multiorgan failure (creatinine 0.87 mg/dL) and hypotension. On clinical examination her abdomen was tender and swollen to palpation without any signs of ongoing peritonitis. An abdominal CT scan revealed a dilated large intestine with parietal pneumatosis from the appendix to the transverse colon associated with extensive pneumoperitoneum (Fig. 1, Fig. 2). The patient was sent to the operating room. Intra operatively we reported the presence of pneumatosis of the right colon and of the right colic flexure and distension of the great omentum (Fig. 3). No resection was needed as normal blood supply to the bowel was present. After surgery the patient was monitored in the intensive care unit and after about 6 h she was transferred to our ward. She had unremarkable recovery. In the fifth day after surgery the leucokytosis was completely solved and we restarted feeding the patient through the PEG. The patient was discharged on the sixth post-operative day.

## 3 Discussion

Pneumatosis coli can be both asymptomatic or life-threatening condition associated to bowel infarction. It can mimic a bowel perforation causing pneumoperitoneum and it could be a misleading indication to surgical exploration especially in the case of uncertain origin of a septic shock.

The appropriate therapy is related to the underlying cause of PCI. The majority of patients without any symptoms are cured without any treatments [21]. Surgery is reserved either for cases of suspected inconvertible intestinal obstruction, for cases of perforation or cases with precancerous conditions [22].

The major etiologic mechanisms of nonsurgical pneumoperitoneum may be grouped under the following categories: postoperatively retained air, thoracic, abdominal, gynecologic, and idiopathic. The most common abdominal cause of nonsurgical pneumoperitoneum is pneumatosis cystoides intestinalis. The condition generally resolves spontaneously but may be indolent and recurrent. Recognition of the potential for non-surgical pneumoperitoneum is important in preventing unnecessary surgical procedures that expose patients to infection, complications, and extended recovery periods. Consideration should be made for close evaluation of radiologic findings in cases where a clear surgical cause of pneumoperitoneum does not exist, and evaluation of other potential causes undertaken. For the case of the surgeon who elects to take a patient for exploratory laparotomy and finds no evidence supporting a surgical etiology, it is acceptable to terminate surgical exploration after adequate inspection of the entire length of the small and large bowel. The recognition of non-surgical pneumoperitoneum at the bedside and further insight into its etiopathogenesis will likely lead to improved morbidity and mortality [24]. With a relevant etiologic condition, attempted conservative management is appropriate in the absence of peritonitis. However, cases of pneumatosis cystoides intestinalis in immunosuppressed patients that evolved rapidly into enteric infection, bowel ischemia, and death have been reported [23,24].

Long term outcomes in non-operative management of pneumoperitoneum caused by PCI aren’t available in current literature.

In our case all the signs and symptoms that led to a suspicion of septic shock caused by intestinal perforation were ascribed to epileptic seizures and dehydration (only by infusing the saline solution during the surgical intervention, lactates decreased from 98 to 23 mg/dL). The clinical exam of abdomen, apparently negative, and the absence of abdominal or digestive signs before admission (the patient was hospitalized for an epileptic seizure – a chest X-ray revealed incidentally the pneumoperitoneum) didn’t support the suspicion of abdominal origin of the sepsis.

## 4 Conclusion

This patient presented a nonsurgical pneumoperitoneum, but it was difficult to understand the fact that the surgical intervention was unneeded in this case because of the unconsciousness of the patient and the impossibility to get a complete anamnesis. The clinical exam of the abdomen was apparently negative, but the laboratory tests showed severe leukocytosis and acidosis and the vital parameters showed hypotension. In addition, the TC scan documented the presence of pneumoperitoneum. Because of all these findings, we first thought to a septic state, due to a bowel perforation. Intra-operatively we found out the presence of pneumatosis of the right colon and of the right colic flexure and distension of the great omentum, with no signs of bowel ischemia or perforation, so no resection was needed. The signs and symptoms that led to a suspicion of septic shock caused by intestinal perforation were ascribed to epileptic seizures and dehydration.

This case highlights the importance of careful consideration of clinical and radiographic findings in the diagnostic and therapeutic approach to pneumoperitoneum. The surgeon has to consider all the potential causes of non-surgical pneumoperitoneum in his decision-making process for surgical versus non-surgical management [25,26].

## Sources of funding

None.

## Ethical Approval

No need of ethical approval.

## Consent

Author obtained the consent for publication from the patient’s kin.

## Author contribution

EB was responsible for the surgical intervention. LC and MR conceived the case report. MR, LC and GP researched and drafted the manuscript. CG, MDD, FB, EB and PC revised the manuscript. All authors read and approved the final manuscript.

## Registration of Research Studies

NA.

## Guarantor

Luigi Conti.

## Provenance and peer review

Not commissioned, externally peer-reviewed.

## Conflicts of interest

None.
